# Plant-derived nodule-specific cysteine-rich peptides as potent antifungal agents against *Cryptococcus neoformans*: mechanisms of action, chimeric peptide enhancement, and immunomodulatory effects

**DOI:** 10.1016/j.crmicr.2025.100407

**Published:** 2025-05-23

**Authors:** Bettina Szerencsés, Csaba Papp, Alexandra Pál, Sándor Jenei, Nelli Németh, Csaba Vágvölgyi, Ferhan Ayaydin, Gabriella Endre, Éva Kondorosi, Ilona Pfeiffer

**Affiliations:** a Department of Biotechnology and Microbiology, Faculty of Science and Informatics, University of Szeged, Szeged, Hungary; b Institute of Plant Biology, HUN-REN Biological Research Centre, Szeged, Hungary; c Functional Cell Biology and Immunology Advanced Core Facility (FCBI-ACF), Hungarian Centre of Excellence for Molecular Medicine (HCEMM), University of Szeged, Szeged, Hungary

**Keywords:** Antimicrobial peptide, Antifungal activity, *Cryptococcus neoformans*

## Abstract

The basidiomycetous yeast *Cryptococcus neoformans* is classified among the four critical fungal pathogens due to its capability of inducing life-threatening meningitis in immunocompromised individuals, particularly AIDS patients. The increasing prevalence of antifungal resistance and limitations of current treatments highlight the urgent need for novel therapeutic strategies. Antimicrobial peptides (AMPs), including plant-derived nodule-specific cysteine-rich (NCR) peptides, offer promising alternatives due to their broad-spectrum activity, multiple cellular targets, and minimal cytotoxic effects on mammalian cells. The aim of this study was to evaluate the anti-cryptococcal efficacy of NCR247, NCR335, NCR169C derivatives, and three synthetic chimeric peptides. Fifteen peptide derivatives and all three chimeras exhibited potent antifungal activity while demonstrating negligible cytotoxicity against murine macrophages. Among them, the X1-NCR247C chimera was the most effective, acting rapidly at low concentrations. Notably, its attachment to the yeast cells augmented the uptake of the cells by murine macrophages, suggesting that in addition to their direct fungicidal effects, antimicrobial peptides can intensify the immune response. These findings underscore the potential of NCR peptide derivatives as anti-cryptococcal agents and highlight the advantages of chimera peptides in improving therapeutic efficacy.

## Highlights

• Nodule-specific cysteine-rich peptide derivatives showed anti-cryptococcal activity.
• The tested peptides exhibited minimal toxicity against murine macrophages.
• X1-NCR247C chimera was the most effective, it acted rapidly at low concentration.
• The X1-NCR247C augmented the uptake of yeast cells by macrophages.
• Fungal cell surface has important role in anchoring the peptides.

## Graphical abstract

**Figure:**
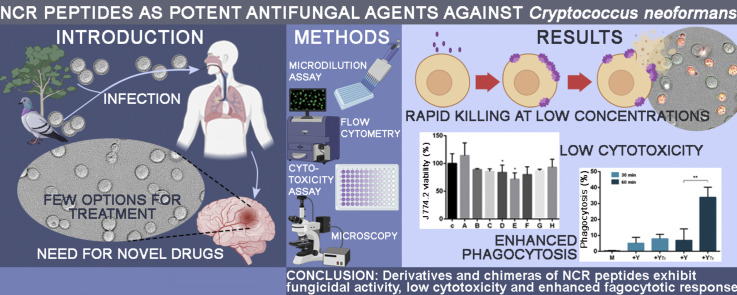

## 1 Introduction

*Cryptococcus neoformans* is a globally distributed, saprophytic yeast that has been classified as one of the four "critical" fungal pathogens by the World Health Organization’s (WHO) Fungal Priority Pathogens List (Sati et al., 2022). This seemingly contradictory classification is due to its opportunistic nature, as it can cause a life-threatening disease called cryptococcosis, primarily in individuals with compromised immune systems, particularly those infected with HIV (Rajasingham et al., 2022). Over the past few decades, cryptococcosis has emerged as a global health problem, due to the increasing number of susceptible individuals. While the incidence of HIV-associated cryptococcosis has decreased during the period of 2014–2020 due to the widespread implementation of effective antiretroviral therapy in AIDS treatment, cases of non-HIV-associated cryptococcal meningitis have risen, with mortality rates ranging from 41 % to 61 % (Zhao et al., 2023). The infection begins with the inhalation of basidiospores or desiccated cells. Although primary infections are often asymptomatic, *C. neoformans* can colonize the lungs, leading to pulmonary cryptococcosis. Innate immune cells, particularly macrophages and dendritic cells, play a crucial role in the host defence by engaging with *Cryptococcus* cells within the pulmonary system. Macrophages can eradicate the yeast cells, but *C. neoformans* has evolved mechanisms to persist within these immune cells, sometimes escaping through non-lytic exocytosis. Consequently, macrophages play a pivotal role in determining the outcome of cryptococcal infection (Nelson et al., 2020). In systemic infection, the yeast cells can spread hematogenously, cross the blood-brain barrier, and establish cryptococcal meningitis — the most common and severe form of the disease (Maziarz and Perfect, 2016). Additionally, *C. neoformans* can cause primary cutaneous cryptococcosis if fungal cells or spores enter the body through open wounds (Du et al., 2015). Several virulence factors contribute to *C. neoformans* pathogenesis, including a polysaccharide capsule, enabling evasion of phagocytosis, and cell wall-located melanin, which provides protection against oxidative stress within the phagolysosome (Zaragoza, 2019).

The current first-line treatment for cryptococcosis is amphotericin B, often in combination with 5′-fluorocytosine for severe cases (Iyer et al., 2021). However, due to the high prevalence of primary resistance and limited availability of 5′-fluorocytosine, particularly in the most affected areas, a combination of amphotericin B and fluconazole is recommended and has shown similar effectiveness. Unfortunately, the emergence of fluconazole and 5′-fluorocytosine resistant strains further complicates the therapy (Spadari et al., 2020) and prolonged usage of amphotericin B can have severe side effects (Laniado-Laborin and Cabrales-Vargas, 2009). These challenges underscore the urgent need for new antifungal agents that are more effective, reduce treatment duration, and mitigate resistance development.

Antimicrobial peptides (AMPs) have emerged as promising alternatives due to their broad-spectrum activity, multiple intracellular targets, and low likelihood of resistance development (Buda De Cesare et al., 2020; Zhang et al., 2021). AMPs are mainly cationic, amphipathic peptides, allowing them to interact with negatively charged macromolecules. Their primary mode of action involves targeting the plasma membrane of susceptible cells, disrupting membrane integrity, and causing ion leakage (Zhang et al., 2021). Plants serve as a vast reservoir of AMPs, including defensins, which are characterized by eight or ten conserved cysteine residues (Carvalho Ade and Gomes, 2009). Legumes within the Inverted Repeat Lacking Clade (IRLC) produce a unique group of defensin-like peptides known as nodule-specific cysteine-rich (NCR) peptides during their symbiotic interaction with bacteria (Maroti et al., 2015). *Medicago truncatula*, a member of the IRLC, encodes over 700 NCR peptides that are predominantly cationic, ranging from 24 amino acids (NCR247) to 64 amino acids (NCR335) in length (Montiel et al., 2017). Despite their sequence diversity, all NCR peptides share four or six conserved cysteine residues essential for disulphide bridge formation, stabilizing their structure.

At low concentrations, NCR peptides promote bacterial differentiation within root nodules, whereas at high concentrations, they exhibit strong antibacterial activity *in vitro*. Their primary mode of action involves membrane interaction, disrupting the integrity and diminishing membrane potential (Tiricz et al., 2013). Notably, NCR247 can also penetrate cells and inhibit bacterial division by interacting with cytosolic proteins such as FtsZ and ribosomal proteins, thereby blocking essential cellular functions (Farkas et al., 2014; Tiricz et al., 2013). Recent studies have shown that the efficacy and spectrum of NCR peptides can be enhanced through the creation of chimeric peptides (Jenei et al., 2020).

Beyond their antibacterial effects, NCR peptides also display antifungal activity. Several highly cationic (isoelectric point, pI > 9.5) NCR peptides *e.g.* NCR247 and NCR335 have demonstrated antifungal activity against human pathogenic *Candida* species like *Ca. albicans, Ca. glabrata, Ca. krusei*, and *Ca. parapsilosis*. Their minimum inhibitory concentration (MIC) varied in range from 1.42 to 10.5 µM, what was comparable to that of amphotericin B (Ordogh et al., 2014). Additionally, fragments of NCR335 have shown inhibitory effects against *Candida* species, including biofilm formation and morphological transitions in dimorphic species (Szerencses et al., 2021). Similarly, the cationic C-terminal fragment of NCR169 and its derivatives have exhibited significant anti-*Candida* properties (Szerencses et al., 2021). Importantly, none of these peptides exhibited cytotoxic effects on human epithelial (Ordogh et al., 2014) or keratinocyte cells (Szerencses et al., 2021). Moreover, NCR peptides have demonstrated antifungal activity against filamentous fungi. For instance, NCR044 effectively inhibits plant pathogenic fungi such as *Botrytis cinerea* and multiple *Fusarium* species (Velivelli et al., 2020).

This study investigates the anti-cryptococcal potential of 20 NCR peptides, including derivatives of NCR169, NCR247, NCR335, which have shown anti-*Candida* activity in previous experiments, as well as recombinant peptides against *C. neoformans* IFM 5844. Our findings reveal that fifteen peptides exhibit fungicidal activity, with recombinant peptides showing the highest potency and MIC values lower than amphotericin B. Additionally, we explore the role of fungal cell walls in anchoring NCR peptides and their effects on murine macrophages, demonstrating low cytotoxicity and an enhanced phagocytic response. By evaluating the therapeutic potential of NCR peptides and engineered chimeras, this study contributes to the development of novel antifungal strategies that may improve treatment outcomes for cryptococcosis.

## 2 Materials and methods

### 2.1 NCR peptides and derivatives

Peptides used in this work are listed in the Supplementary Table 1 together with their main physico-chemical properties. Peptides were synthesized with C-terminal amidation via standard procedure of the solid-phase peptide synthesis (SPPS) by using an automatic peptide synthesizer (CEM Liberty Blue) and purified by reverse-phase high-performance liquid chromatography (RP-HPLC) according to the previously published method (Jenei et al., 2020; Szerencses et al., 2021).

### 2.2 Cultivation conditions

*C. neoformans* strain IFM 5844 was cultured overnight in YPD medium (1 % pepton, 1 % dextrose, 0.5 % yeast extract) at 30 °C in water bath shaker. The cells were harvested by centrifugation (10 min, 5000 g) washed twice in sterile distilled water and suspended in the appropriate medium. The cell number was determined in Bürker chamber and adjusted to the desired concentration for further experiments.

Mouse macrophage J774.2 cell lines were maintained and cultivated in DMEM medium (Dulbecco’s Modified Eagle’s Medium, Biosera, Cholet, France) supplemented with 10 % fetal bovine serum (FBS, Capricorn Scientific GmbH, Ebsdorfergrund, Germany) and 1 % Penicillin/Streptomycin solution (Capricorn Scientific GmbH, Ebsdorfergrund, Germany). The cells were cultivated at 37 °C under 5 % CO_2_ level and 95 % relative humidity.

### 2.3 Determination of the minimal inhibitory and the fungicide concentration of the peptides

The susceptibility of *Cryptococcus neoformans* IFM 5844 to 20 peptides (Supplementary Table 1) was tested in a widely used synthetic medium to cultivate yeasts (YNB) and in a serum-free medium what is able to support the proliferation of macrophage cells (X-VIVO).

YNB medium: five-fold diluted Difco Yeast Nitrogen Base w/o Amino Acids medium (Becton, Dickinson and Company, Sparks, MD, USA) supplemented with 1 % dextrose

X-VIVO medium: half-diluted X-VIVO™ 15 cell culture medium (Lonza, Verviers, Belgium).

The minimal inhibitory concentration (MIC) of each peptide was determined by using micro-dilution assay. The *C. neoformans* suspensions were adjusted to 5 × 10^4^ cells/mL concentration in either YNB or X-VIVO medium. A 96-well microtiter plate was prepared by adding 95 µL suspension per well followed by 5 µL of serially two-fold diluted peptide solutions achieving a final volume of 100 µl. The concentration of the peptides ranged from 25 to 0.39 µM. For control samples, 5 µL pure medium was added to the yeast cell suspension instead of peptides. Plates were incubated at 37 °C for 48 h in X-VIVO medium and 30 °C for 72 h in YNB medium. After incubation, optical density was measured at 620 nm using a SPECTROstar Nano plate reader (BMG LabTech, Offenburg, Germany). MIC was defined as the lowest peptide concentration that resulted in ≥ 90 % growth inhibition, relative to untreated controls. The experiments were carried out in three independent biological replicates.

Minimal fungicidal concentration (MFC) determination. To assess fungicidal activity, both control and peptide-treated cultures from the MIC assays were diluted 10-fold and 100-fold in sterile distilled water. Five µL from each dilution was then spotted onto solid YPD medium (1 % pepton, 1 % dextrose, 0.5 % yeast extract, 2 % agar). The plates were incubated at 30 °C for 48 h and fungal growth was evaluated. MFC was defined as the lowest peptide concentration at which no visible fungal growth was detected, indicating complete fungicidal activity.

### 2.4 Characterization of the short-term sub-MIC effects of peptides

To assess the time-dependent fungicidal activity of X1-NCR247C, *C. neoformans* cells cultivated for 16 h at 30 °C were collected by centrifugation and washed twice with sterile distilled water. The cell concentration was adjusted to 2 × 10^6^ cells/mL in YNB medium and treated with 0.19 µM X1-NCR247C (half-MIC) for 5, 20 and 60 min. Following treatment, cells were pelleted by centrifugation washed with distilled water and serially diluted 10-fold and 100-fold. Fifty microliters of each dilution were plated onto solid YPD medium in triplicate.

To evaluate the concentration-dependent effects of X1-NCR247C, *C. neoformans* cells (2 × 10^6^ cells/mL) were treated for 30 min with various peptide concentrations (0.19 µM – half-MIC, 0.1 µM, 0.05 µM). After treatment, the cells were pelleted by centrifugation, washed with distilled water and serially diluted 10-fold and 100-fold. Fifty microliters of each dilution were spread onto solid YPD medium in triplicate. For both experimental setups colony forming unit (CFU) counts were determined after 48-hours incubation at 30 °C. The CFU values of peptide-treated samples were compared to untreated control samples, providing insights into the extent of fungal viability reduction. Three independent biological replicates were performed.

### 2.5 Cell membrane permeability assay

The effect of the peptides on the cell membrane permeability of *C. neoformans* IFM 5844 cells was checked by flow cytometry with calcein-AM assay (Olson, et al., 2001). Calcein-acetoxymethyl ester (calcein-AM) is a membrane-permeable live-cell dye that, upon entering the cell, is cleaved by intracellular esterases, converting it into calcein, a membrane-impermeable fluorescent marker. The assay was carried out by suspending a total of 1 × 10^7^ cells in 100 µl YNB medium containing 20 µg/mL indomethacin (Sigma-Aldrich, Saint Louis, MO, USA) and incubating them at 30 °C for 30 min to inhibit calcein transporters. Following this pre-treatment, membrane-permeable calcein-AM (Invitrogen, Waltham, MA, USA) was added to a final concentration of 5 µg/mL, and the samples were incubated at 30 °C for 3 h allowing the calcein-AM to enter the cells where esterases converted it to fluorescent calcein. After incubation, cells were washed twice with sterile distilled water and subsequently exposed to peptides at half-MIC concentrations in YNB medium for 2 h at 30 °C. Following peptide treatment, cells were washed twice with sterile distilled water, and fluorescence intensity was measured using a FlowSight® flow cytometer (Amnis-EMD Millipore) equipped with a 488-nm excitation laser. Intact cells stained with calcein-AM but not exposed to peptides served as controls. To ensure statistical reliability, four independent experiments were conducted, and fluorescence intensity was recorded from 10,000 cells per sample during each experiment.

### 2.6 Cytotoxicity assessment of peptides on mouse macrophage J774.2 cell line

J774.2 cells were cultured as described previously and seeded into microtiter plates at a density of 5 × 10⁴ cells per well. The cells were incubated at 37 °C under 5 % CO₂ and 95 % relative humidity in X-VIVO™ medium until they adhered to the plate surface. Following attachment, cells were treated with peptides at their respective MIC concentrations in X-VIVO medium for 3 h under the same incubation conditions.

After peptide treatment, cells were washed with PBS (phosphate-buffered saline) and incubated in X-VIVO™ medium supplemented with 0.5 mg/mL MTT reagent (3-[4,5-dimethylthiazol-2-yl]−2,5-diphenyltetrazolium bromide, Merck KGaA, Darmstadt, Germany). The MTT assay was used to evaluate cell viability by staining live cells, leading to the formation of formazan crystals. These crystals were dissolved in dimethyl sulfoxide (DMSO), and absorbance was measured at 570 nm using a SPECTROstar Nano plate reader (BMG LabTech, Offenburg, Germany). The absorbance of untreated control cells was set as 100 % viability, and experimental results were expressed relative to this control. Each experiment was conducted in at least three independent biological replicates to ensure statistical reliability.

### 2.7 Phagocytosis assay on peptide-treated *C. neoformans* cells by mouse macrophages

*C. neoformans* was cultivated for 16 h at 30 °C, collected by centrifugation (5000 g, 5 min), and washed twice with sterile distilled water and once with PBS. The cell suspension was adjusted to 2.5 × 10⁶ cells/mL, followed by treatment with X1-NCR247C peptide at 3.12 µM (MIC) in X-VIVO™ medium. After 15 min of incubation at 30 °C, the peptide-containing solution was removed by centrifugation, and the *C. neoformans* cells were washed with PBS and re-suspended in 100 µL PBS. Untreated *C. neoformans* cells served as controls.

To label *C. neoformans* cells, the suspension was supplemented with 11 µL of 1 M Na₂CO₃ (pH 10) and 2 µL of Alexa Fluor 488-labelled-concanavalin (Invitrogen, Waltham, MA, US), followed by incubation at room temperature for 30 min, protected from light. After PBS washing, the cell density was determined using a Bürker chamber and adjusted to the required concentration.

Macrophages, cultured as previously described, were infected at a 5:1 ratio (*C. neoformans* to macrophages), where 5 × 10⁴ macrophages/100 µL were incubated with 2.5 × 10⁵ labelled *C. neoformans* cells/100 µL in X-VIVO™ medium. The infection was carried out in three biological repeats, each with two technical replicates at 37 °C under 5 % CO₂ and 95 % relative humidity for 30 and 60 min.

Following incubation, cells from each technical replicate were collected by centrifugation (200 g, 10 min), washed once with PBS, and re-suspended in 100 µL PBS buffer for detection. A random selection of 1000 macrophages was evaluated in each experimental sample, and the proportion of macrophages harboring fluorescently labelled *C. neoformans* cells was quantified utilizing imaging flow cytometry (FlowSight®, Amnis-EMD Millipore) equipped with a 488-nm excitation laser, with data analysis conducted through the IDEAS software platform.

### 2.8 Susceptibility of *C. neoformans* cells and protoplasts to peptides

The *C. neoformans* IFM 5844 strain was grown in YPD nutrient solution at 30 °C for 12 h by vigorous shaking until the exponential phase was reached (about 5 × 10^7^ cells/mL).

For protoplast preparation cells were harvested by centrifugation and re-suspended in 20 mL of pre-treatment buffer (10 mM Tris–HCl, pH 9; 5 mM EDTA-Na_2_) supplemented with 0.1 % 2-mercaptoethanol. After incubation at room temperature for 20 min, the samples were centrifuged and the cell pellet was washed with 0.8 M sorbitol solution. The pellet was then re-suspended in 20 mL of protoplasting solution (prepared by dissolving 1 g lyophilised *Helix pomatia* digestive enzyme in 100 mL sorbitol) and the suspension was incubated for 60 min at room temperature. Protoplast formation was monitored every 30 min under bright field microscope. Once formed, protoplasts were collected by centrifugation, washed twice with 0.8 M sorbitol, and their concentration was determined in a Bürker chamber.

To assess peptide susceptibility, 2 × 10^6^ protoplasts and the same number of intact cells were treated separately with X1-NCR247C peptide at 0.78 or 0.39 µM in YNB supplemented with 0.8 M sorbitol for 30 min at 30 °C. (The MIC value of X1-NCR247C peptide in this solution was 1.56 µM.) Following incubation, cells and protoplasts were washed once with 0.8 M sorbitol to remove residual peptide, and 10-fold and 100-fold dilutions were prepared. Fifty microliters from each dilution were mixed into 2 mL of top-agar (0.8 M sorbitol with 1 % agar) and spread onto the surface of YPD solid medium supplemented with 0.8 M sorbitol. Plates were incubated for 3 days at 30 °C, after which colony forming units were counted to determine the survival rate of peptide-treated cells and protoplasts.

### 2.9 Confocal microscopy imaging to identify the localisation of peptides

*C. neoformans* IFM 5844 was cultured overnight in YPD medium at 30 °C in water bath shaker. The cells were harvested by centrifugation (10 min, 5000 g) washed twice in sterile distilled water and suspended in PBS buffer. The cell concentration was determined in Bürker chamber and adjusted to 1 × 10^7^ cells/mL. To visualize peptide localization, cells were treated with 1 µM fluorescein-isothiocyanate (FITC)-conjugated NCR335 peptide in YNB for 5 min at room temperature. Following incubation, fluorescence detection was performed using a Leica Stellaris 5 confocal laser scanning microscope (Leica Microsystems GmbH) with 63x oil immersion objective (N.A. 1.40) using dye configuration preset for FITC imaging.

### 2.10 Effect of X1-NCR247C peptide and calcofluor white on *C. neoformans* cells

To assess the effect of X1-NCR247C peptide and calcofluor white, *C. neoformans* cells were subjected to different treatments. For peptide exposure, cells were incubated in YNB medium with X1-NCR247C at its MIC (0.39 µM) for 20 min. For staining, cells were treated with calcofluor white at 2.5 µg/mL for the same duration. As a control, cells were incubated only with calcofluor white for 20 min.

For combined treatment, cells were first exposed to calcofluor white (2.5 µg/mL) for 5 min, followed by the addition of X1-NCR247C (0.39 µM), and the incubation continued for 15 additional minutes. Following treatment, cells were washed once with sterile distilled water to remove residual peptide and dye. Serial 10-, 100-, 1000-, and 10,000-fold dilutions were prepared, and 50 µL from each dilution was plated onto solid YPD medium in triplicate. After 48 h of incubation at 30 °C, colony-forming units were counted to determine cell viability under each condition.

### 2.11 Statistical analysis

Data were expressed as the mean ± standard deviation (SD), derived from a minimum of three independent experiments. Statistical analysis was conducted utilizing GraphPad Prism version 6.07 (GraphPad Software, Inc., La Jolla, *CA*, USA). Unpaired *t*-test was used; results were considered statistically significant when *p* ≤ 0.05.

## 3 Results

### 3.1 Anti-cryptococcal effect of peptides and their derivatives

The anti-cryptococcal activity of 20 peptides, derivatives and recombinant peptides originating from three *M. truncatula* NCR peptides NCR169, NCR335 and NCR247, was tested against *C. neoformans* IFM 5844 (Supplementary Table 1). Among the tested peptides, three were derived from NCR169, specifically the highly cationic C-terminal region in its linear (NCR169C_17–38_) and oxidized (NCR169C_17–38_ox) forms, along with a modified variant where two tryptophan residues (W10 and W20) were substituted with alanine (NCR169C_17–38_W_10,20_/A). Additionally, eleven short derivatives of the 64-amino-acid-long NCR335 peptide were included, with four originating from the N-terminal region and seven from the C-terminal region. One derivative (NCR247C) of NCR247, the shortest NCR peptide with notable antimicrobial activity (Tiricz et al., 2013), was also tested.

Three recombinant peptides were selected upon their known antibacterial effect (Jenei et al., 2020), all incorporating the NCR247C fragment in various configurations. These included the X1-NCR247C chimera which fused the N-terminal fragment of NCR335 (NCR335N_7–19_, referred to as X1) with NCR247C, and two additional chimeric peptides, X2-NCR247C and NCR247C-X2, both of which contained the 11-amino-acid-long KALAALAKKIL sequence (designated as X2) in both combinations. The X2 sequence originates from TP10 (Transportan 10), a 21-amino-acid-long cell-penetrating cationic peptide (Yandek et al., 2007), which was also included in the study. To assess antifungal potency, the minimum inhibitory concentration (MIC) of each peptide was determined using a microdilution assay in YNB medium, with two-fold serial dilutions ranging from 25 µM to 0.39 µM. Fifteen out of the 20 peptides displayed antifungal activity against *C. neoformans* (Table 1). The peptides exhibiting the highest efficacy were the chimeric peptides X1-NCR247C (MIC: 0.39 µM), NCR247C-X2 and X2-NCR247C (MIC: 0.78 µM).

**Table 1:** Minimal inhibitory concentration (MIC in µM) of the peptides against *Cryptococcus neoformans* IFM 5844 strain in X-VIVO and YNB medium.

Ĉ indicates two cysteines joined by disulphide bridge.

„-„ no inhibitory effect was detected in the tested concentration range.

ND: not determined.

Among the NCR169-derived peptides, NCR169C_17–38_ and its oxidized form NCR169C_17–38_ox exhibited relatively high efficiency (MIC: 3.12 µM), while the tryptophan-to-alanine variant (NCR169C_17–38_W_10,20_/A) showed slightly reduced activity (MIC: 6.25 µM).

Similar activity was observed for the NCR335 peptide (MIC of 3.12 µM) albeit notable variances were recorded in the efficacy of its derivatives. Among the N-terminal fragments, NCR335N_1–15_ exhibited antifungal effects comparable to NCR335 (MIC: 6.25 µM), while the extended version, NCR335N_1–19_, showed markedly reduced activity (MIC: 25 µM). Other N-terminal derivatives exhibited varying degrees of activity: NCR335N_7–21_ had moderate efficacy (MIC: 12.5 µM), whereas the 14 amino acid-long NCR335N_16–29_ possessing a net charge of +4.1 was ineffective.

The C-terminal part of NCR335, NCR335C_1–33,_ with the characteristic pattern of cysteine residues proved effective (MIC: 6.25 µM) as were its truncated versions; NCR335C_13–33_ maintained similar activity (MIC: 6.25 µM), whereas NCR335C_17–33_ exhibited even higher potency (MIC: 3.12 µM), aligning with the full-length NCR335 peptide. Conversely, the four short derivatives of NCR335 (NCR335C_17–27_, NCR335C_1–8_, NCR335C_9–16_ and NCR335C_1–16_) lacked inhibitory effect on the studied strain.

The NCR247 peptide and its C-terminal derivative (NCR247C) showed identical inhibitory activity with MIC value of 6.25 µM suggesting that truncation of the peptide did not compromise its antifungal potency. Regarding the X2 sequence found in the chimeric peptides, this short fragment alone did not exhibit antifungal activity within the tested concentration range. However, its parent peptide, TP10, was active against *C. neoformans* (MIC: 12.5 µM).

To compare the effectiveness of the peptides with established anti-cryptococcal agents the MIC values of amphotericin B and fluconazole were determined. For *C. neoformans* IFM5844, the MIC of amphotericin B was 1.56 µM, which value is higher than that of the three chimeric peptides (X1-NCR247C, NCR247C-X2 and X2-NCR247C) indicating their superior potency. Fluconazolewas notably less effective against *C. neoformans* under these conditions (MIC: 12.50 µM), in comparison to fourteen peptides that exhibited equal or greater efficacy (Table 1).

Since subsequent experiments were to be conducted in X-VIVO medium, it was necessary to reassess peptide activity, as AMPs can exhibit variable antimicrobial effects depending on the medium used (Farkas et al., 2018). The MIC values of peptides exhibiting activity in YNB medium were re-evaluated in X-VIVO medium (Table 1). Under these conditions, the three chimeric peptides maintained their superior performance, although with slightly elevated MIC values, NCR247C-X2 at 1.56 µM, while X1-NCR247C and X2-NCR247C at 3.12 µM. Among the NCR335 derivatives, only the three active N-terminal fragments (NCR335N_1–15_, NCR335N_1–19_, NCR335N_7–21_) retained their antifungal activity at the same MIC values as in YNB medium. All other peptides lost their activity in the tested concentration range in X-VIVO medium (Table 1).

### 3.2 Peptide-mediated inhibition is attributed to fungicidal activity

To determine whether the inhibitory effect of the peptides against *C. neoformans* was due to fungistatic or fungicide mode of action, the viability of peptide treated cells was assessed. All tested peptides (NCR335N_1–19_, NCR335N_1–15_, NCR335N_7–21_, NCR335C_1–33_, NCR335C_13–33,_ NCR335C_17–33,_ NCR169C_17–38_, NCR169C_17–38_ox, NCR169C_17–38_W_10,20_/A, NCR247, NCR247C, X1-NCR247C, NCR247C-X2, X2-NCR247C and TP10) exhibited a fungicidal effect, effectively killing yeast cells (Supplementary Fig. 1). The fungicide concentrations required for the complete elimination of the *Cryptococcus* cells were equal to their minimum inhibitory concentrations in the tested conditions.

**Fig. 1:**
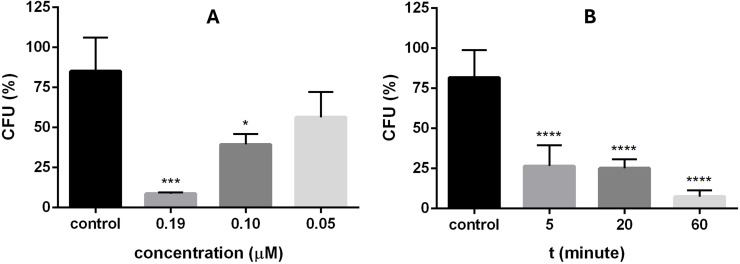
Concentration- (A) and time- (B) dependent activity of X1-NCR247C on *C. neoformans* IFM5844. (A) Cells were exposed to X1-NCR247C at sub-MIC (0.19, 0.1 and 0.05 µM) for 30 min. Then the number of CFU was determined, which is given relative to the control as CFU ( %). (B) Time dependent activity was checked by treatment of the cells with 0.19 µM peptide for 5, 20 and 60 min. (*: *p* ≤ 0.05, ***: *p* ≤ 0.001, ****: *p* ≤ 0.0001, unpaired *t-*test).

To further characterize the short-term, sub-MIC effect of the peptides, X1-NCR247C (the most potent peptide with the lowest MIC) was selected for additional analysis. The viability of *C. neoformans* IFM 5844 cells was assessed after 30 min treatment with the X1-NCR247C peptide at half-MIC (0.19 µM) and lower concentrations (0.1 and 0.05 µM). Additionally, the impact of time-dependent exposure was assessed by treating cells with X1-NCR247C at half-MIC for 5, 20, and 60 min. Following treatments, colony-forming units were quantified and compared to untreated control samples to determine relative CFU percentages (Fig. 1). Our findings demonstrate that X1-NCR247C exerts a potent killing effect even at sub-MIC concentrations (Fig. 1**A**). A significant reduction in colony-forming units was observed within 30 min of exposure, with a highly significant decrease (*p* ≤ 0.001) at half-MIC and a still notable reduction (*p* ≤ 0.05) at quarter-MIC. Within just 5 min of treatment at half-MIC, the proportion of viable cells dropped to 25 %, and after 60 min of exposure, only 5 % of the cells remained viable (Fig. 1**B**).

### 3.3 Peptide localization on *C. neoformans* cell surface and its impact on membrane permeability

**Fig. 2:**
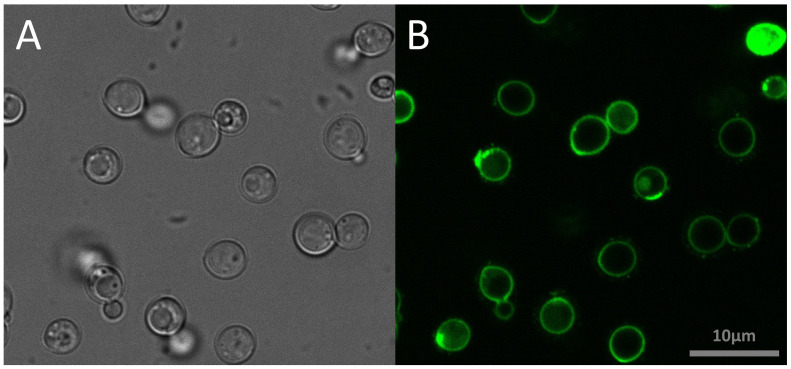
Localisation of FITC-labelled NCR335 peptide in *C. neoformans* IFM 5844 cells **A**: bright field, **B**: fluorescence microscopy image The experiment was carried out in four biological replicates.

**Fig. 3:**
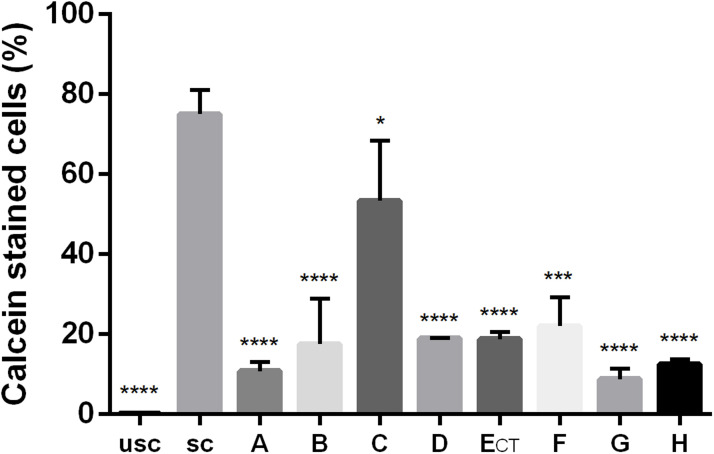
Proportion of *Cryptococcus neoformans* cells stained with calcein in control samples and after peptide treatments. The number of calcein-stained cells was counted by flow cytometry after treatment with half-MIC concentrations of peptides, and plotted as a percentage of the initial cell number. Treatments were as follows: usc: unstained yeast cells, sc: calcein-AM-stained, non-treated yeast cells, A: NCR169C_17–38_, B: NCR169C_17–38_W_10,20_/A, C: NCR335N_7–21_, D: NCR335C_17–33_, E_CT_: NCR247C, F: X1-NCR247C, G: NCR247C-X2, H: X2-NCR247C-treated samples. (*: *p* ≤ 0.05, **: *p* ≤ 0.01, ***: *p* ≤ 0.001, ****: *p* ≤ 0.0001, unpaired *t-*test).

To investigate the distribution of peptides in *C. neoformans* cells, FITC-labelled NCR335 peptide was applied at sub-lethal concentration and visualized using confocal laser scanning microscope. After 5 min of exposure, the peptide accumulated on the cell surface (Fig. 2). Given this surface localization and the rapid fungicidal activity observed, we further examined the effect of peptides on membrane permeability. Membrane permeability was assessed using flow cytometry by measuring the intracellular accumulation of fluorescent calcein in *C. neoformans* cells. The proportion of calcein-stained cells was analysed after two hours of treatment with different peptides at half-MIC concentrations (Fig. 3). In the unstained control group, <0.5 % of cells showed fluorescence. In the calcein-AM-stained control group (without peptide exposure), 75 % of cells were fluorescent. Treatment with most of the peptides significantly disrupted membrane integrity, leading to a highly significant reduction in fluorescent cells (*p* ≤ 0.0001). Peptides NCR169C_17–38_, NCR169C_17–38_W_10,20_/A, NCR335C_17–33_, NCR247C, NCR247C-X2 and X2-NCR247C caused the highest calcein leakage, indicating severe membrane permeability disruption (samples A, B, D, E_CT_, G and H in Fig. 3). The NCR247C-X2-treated sample exhibited the lowest proportion of stained cells (8 %), highlighting its strong membrane-disrupting effect. Interestingly, despite having the lowest MIC, X1-NCR247C, had a slightly weaker effect in this assay (sample F in Fig. 3), with a reduction in fluorescent cells at a significance level of *p* ≤ 0.001. These findings suggest that the antifungal peptides localize to the fungal surface, where they rapidly increase membrane permeability, leading to cytoplasmic leakage and cell death. The degree of permeability alteration varies among peptides, correlating with their fungicidal potency.

### 3.4 Intact *C. neoformans* cells are more susceptible to NCR peptides than protoplasts

**Fig. 4:**
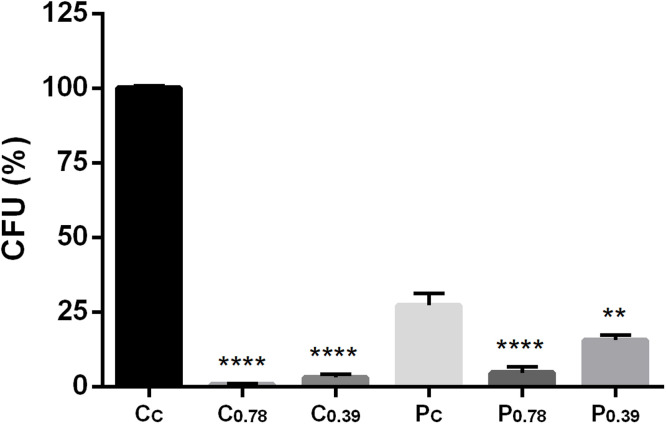
Relative CFU counts of *C. neoformans* IFM **5844 intact cells and protoplasts after exposure to X1-NCR247C (0.78 or 0.39µM) for 30 min.** Following treatment, cells (C) and protoplasts (P) were embedded in topagar and plated onto nutrient rich medium and CFU were counted after 3 days. Controls included untreated intact cells (C_C_) or protoplasts (P_C_). (****: *p* ≤ 0.0001, **: *p* ≤ 0.01, unpaired *t-*test).

Given the observed impact of peptides on cell membrane permeability, we investigated whether the presence of the cell wall influences peptide susceptibility by comparing the effects of X1-NCR247C on *C. neoformans* intact cells and protoplasts. Cells and protoplasts were exposed to X1-NCR247C at sub-MIC concentrations (0.39 and 0.78 µM) for 30 min, after which the CFU count was assessed relative to untreated controls. Peptide treatment led to a statistically significant reduction in the viability of both intact cells and protoplasts, but a notable difference in sensitivity was observed (Fig. 4). At 0.78 µM, CFU counts were reduced by 99 % in intact cells and 83 % in protoplasts, indicating strong fungicidal activity in both conditions. However, at the lower concentration of 0.39 µM, intact cells still exhibited a 97 % reduction, whereas the reduction in protoplasts was significantly lower (43 %). These results suggest that intact cells are more susceptible to peptide-mediated killing than protoplasts, implying a potential role of the cell wall in facilitating peptide interaction or activity. This trend was further confirmed using two additional peptides, NCR335C_17–33_ and NCR335N_16–29_ both of which displayed a similar difference in susceptibility between intact cells and protoplasts (data not shown).

**Fig. 5:**
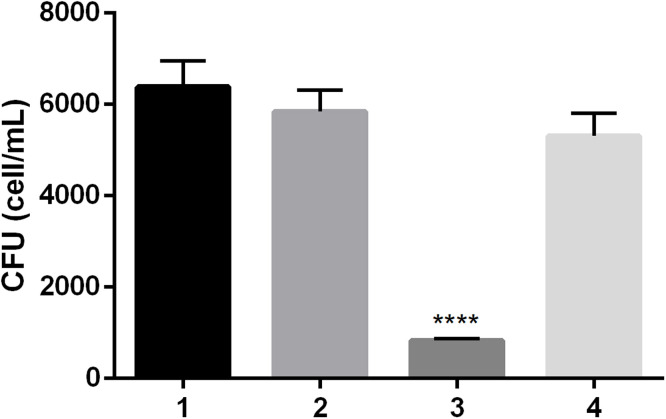
Effect of calcofluor white pre-treatment on *C. neoformans* sensitivity to X1-NCR247C. CFU was counted after (1) no treatment (control), (2) calcofluor white staining for 20 min, (3) X1-NCR247C treatment (0.39 µM, 20 min), (4) calcofluor white pre-treatment (5 min), followed by X1-NCR247C exposure (0.39 µM, 15 min) (****: *p* ≤ 0.0001, unpaired *t*-test).

To further explore the role of the cell wall in mediating peptide activity we conducted experiments using calcofluor white a fluorescent dye that binds chitin - a major component of the fungal cell wall. Control experiments were conducted by separately incubating yeast cells with calcofluor white or X1-NCR247C peptide for 20 min, followed by CFU quantification. As expected, calcofluor white alone did not reduce CFU counts, whereas treatment with X1-NCR247C significantly decrease cell viability (*p* ≤ 0.0001) (Fig. 5). In contrast, pre-treating yeast cells with calcofluor white for 5 min before peptide exposure rendered the cells resistant to X1-NCR247C, as no reduction in the number of colony-forming units was observed. These findings indicate that calcofluor white binding to chitin alters the ability of the peptide to exert its antifungal effect, potentially by blocking peptide interaction with the fungal cell wall. This supports the hypothesis that the cell wall plays a crucial role in facilitating the activity of NCR peptides against *C. neoformans*.

### 3.5 Cytotoxicity assessment on J774.2 mouse macrophages confirms the safety of NCR peptides

**Fig. 6:**
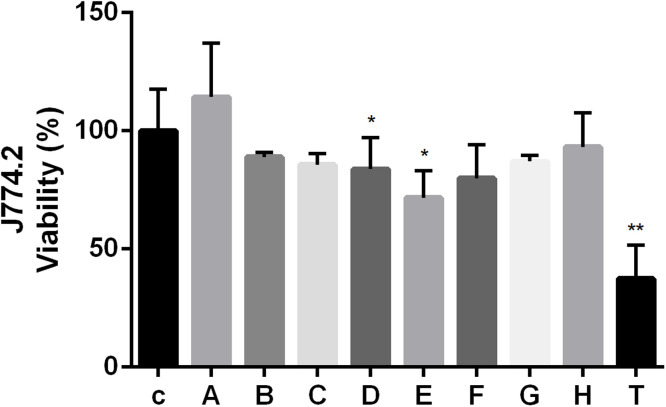
Cytotoxicity of NCR peptides on J774.2 macrophage cells, measured via MTT assay after 3-hour peptide treatment. Peptide concentrations coincide with their MIC value for *C. neoformans* IFM 5844 in X-VIVO medium. c: control (untreated J774.2 cells), A: NCR169C_17–38_, B: NCR169C_17–38_W_10,20_/A, C: NCR335N_7–21_, D: NCR335C_17–33_, E: NCR247, F: X1-NCR247C, G: NCR247C-X2, H: X2-NCR247C, T: TP10. (*: *p* ≤ 0.05, **: *p* ≤ 0.01, unpaired *t*-test).

The potential cytotoxic effects of NCR peptides were evaluated using J774.2 mouse macrophage cells. Macrophage cells were exposed to each peptide for 3 h at concentrations corresponding to their MIC values against the *C. neoformans* IFM 5844 strain in X-VIVO medium (Table 1). The viability of the macrophage cells was assessed using the MTT reduction assay. Results showed that most NCR peptides exhibited no significant cytotoxic effects on J774.2 cells (Fig. 6) However, two peptides, NCR247 and NCR335C_17–33_, both at 25 µM caused a slight reduction in viability (25 % and 15 %, respectively). In contrast, TP10, administered at 25 µM, significantly reduced macrophage viability compared to the untreated control group (*p* ≤ 0.01).

### 3.6 Peptide treatment enhances the phagocytic activity of macrophages against *C. neoformans*

**Fig. 7:**
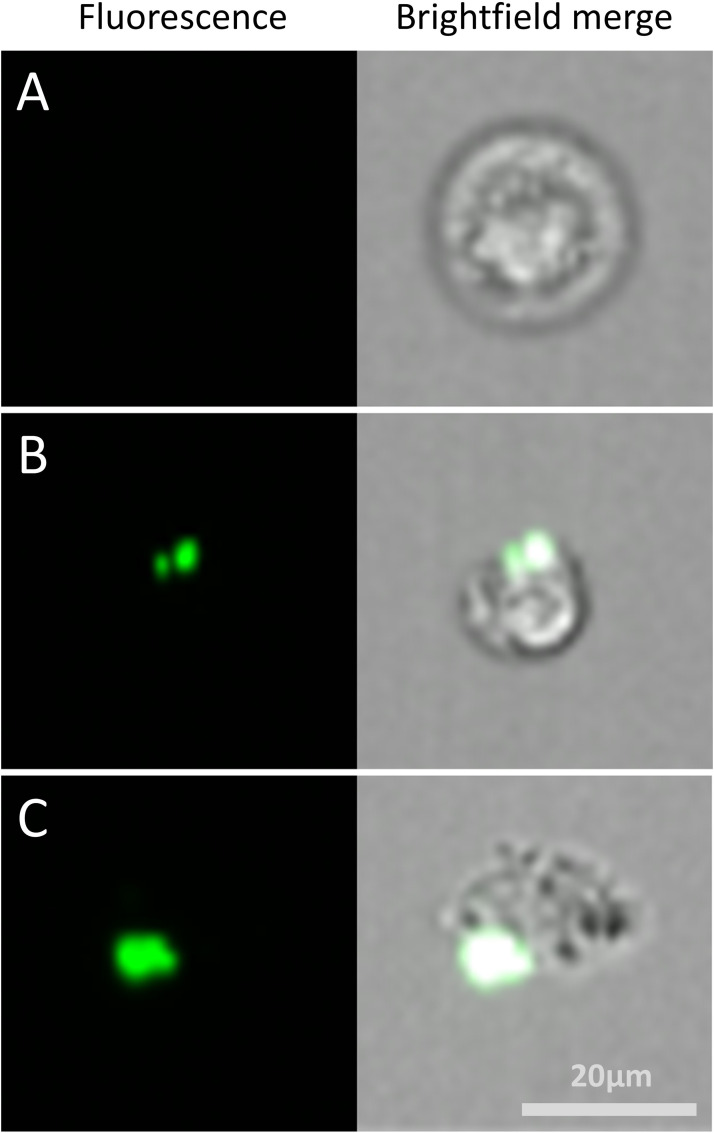
Identification of macrophage - *C. neoformans* interaction 60 min post-infection. **A**: non-infected macrophages, B: macrophages infected with Alexa Fluor 488-concanavalin stained *C. neoformans* cells; C: macrophages infected with Alexa Fluor 488-concanavalin stained and X1-NCR247C-treated *C. neoformans* cells. The images were taken using imaging flow cytometry; the cells illustrated representatives of samples M, +*Y* and +Y_Tr_ (refer to Fig. 8.) at 60-minute post-infection.

**Fig. 8:**
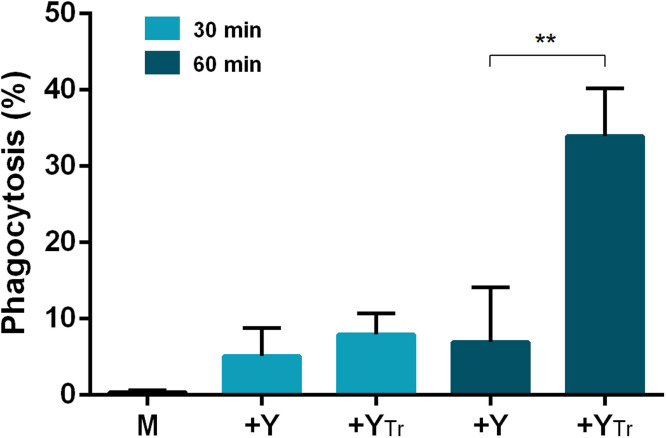
Flow cytometry analysis of macrophage phagocytosis at 30 and 60 min post-infection. M: non-infected macrophages, +Y: Macrophages infected with untreated yeast cells +Y_Tr_: Macrophages infected with X1-NCR247C-treated yeast cells. (**: *p* ≤ 0.01) Light blue columns: 30-minute post-infection, Dark blue columns: 60-minute post-infection.

Since macrophages play a critical role in the eradication of *C. neoformans*, we investigated how peptide treatment influences their phagocytic function. The phagocytosis kinetics of J774.2 mouse macrophages was studied using *C. neoformans* cells labelled with fluorescent dye Alexa Fluor 488-labelled-concanavalin. Macrophages were infected with either untreated or peptide-treated yeast cells at a 5:1 yeast-to-macrophage ratio and incubated prior to staining. For peptide treatment, X1-NCR247C (the most potent peptide with the lowest MIC) was selected and applied at 3.12 µM (MIC in X-VIVO medium). Flow cytometry was used to identify and sort out macrophages displaying a fluorescent signal, which were confirmed as phagocytic macrophages by imaging flow cytometry (Fig. 7). A control experiment was performed to evaluate the natural phagocytic ability of J774.2 macrophages against Alexa Fluor 488-concanavalin stained *C. neoformans* cells. Flow cytometry measurements were conducted at two post-infection time points (30 min, 60 min), quantifying the proportion of macrophages exhibiting a fluorescent signal, which served as an indicator of their phagocytic capacity (Fig. 8). At 30 min post-infection, phagocytosis had already begun, with 5.12 % of macrophages in the control group being identified as phagocytic. In contrast, the percentage of phagocytic macrophages in the X1-NCR247C-treated group was slightly higher (7.95 %). At 60 min post-infection, a significant increase in phagocytosis was observed. In the X1-NCR247C-treated sample, 33.95 % of macrophages exhibited phagocytic activity, a more than fourfold increase compared to 6.95 % in the control group (Fig. 8).

These results indicate that treatment with X1-NCR247C significantly enhances macrophage phagocytosis of *C. neoformans*, suggesting that antimicrobial peptides may boost the immune response by promoting the uptake of fungal cells.

## 4 Discussion

Our study demonstrates the potent antifungal activity of plant-derived NCR peptides against *Cryptococcus neoformans*, a major opportunistic human pathogen (Tugume et al., 2023). Fifteen out of the 20 tested peptides exhibited significant inhibitory effects, with three chimeric peptides (X1-NCR247C, NCR247C-X2, and X2-NCR247C) displaying superior efficacy, surpassing amphotericin B in terms of minimal inhibitory concentration (MIC). Our findings indicate that truncating long peptides, such as the 64-amino-acid cationic peptide NCR335 or the originally non-antifungal NCR169, can yield shorter derivative peptides with retained or even enhanced antifungal activity (Ordogh et al., 2014; Szerencses et al., 2021).

Analysis of NCR335N derivatives revealed that peptides with a net positive charge were more effective. Among these, NCR335N_1–19_, NCR335N_1–15_ and NCR335N_7–21_ showed significant activity, whereas NCR335N_16–29_ was inactive against *C. neoformans*. Similarly, C-terminal derivatives of NCR335 displayed variable activity, with NCR335C_17–33_ exhibiting the highest efficacy, while NCR335C_1–33_ and NCR335C_13–33_ showed reduced activity*.* Further truncation of NCR335C_17–33_ (NCR335C_17–27_) led to a complete loss of activity, indicating that the C-terminal sequence (C_28–33_) has an essential contribution for the antifungal effect of NCR335C_17–33_. Peptides shorter than 12 amino acids or with a net charge lower than +5 were ineffective, emphasizing that both the length and the positive charge are critical determinants of the antifungal potency.

Similarly, NCR169C peptide derivatives (22 amino acids, positively charged) exhibited strong antifungal activity. Both the linear (reduced) and hairpin (oxidized) forms demonstrated equivalent efficacy. However, substituting tryptophan with alanine (NCR169C_17–38_W_10,20_/A) resulted in decreased activity, suggesting a key role for tryptophan residues in antifungal peptide function (Feng et al., 2020).

The C-terminal half of NCR247 (12 amino acids) retained the same activity as the full length peptide, but its effectiveness was further enhanced through chimeric peptide design. The three recombinant chimeric peptides (X1-NCR247C, X2-NCR247C, and NCR247C-X2) exhibited the lowest MIC values surpassing standard antifungals. Interestingly, X2, derived from TP10, was inactive on its own, but when fused to NCR247C, the resulting chimeras displayed significantly enhanced activity. The most potent peptide, X1-NCR247C, was created by fusing NCR247C with a 13-amino-acid segment from NCR335, further highlighting the potential of chimeric peptide engineering for antifungal applications.

Viability assays confirmed the fungicidal nature of NCR peptides, showing a rapid reduction in *C. neoformans* viability, even at sub-MIC concentrations. Unlike fungistatic agents, these peptides actively kill fungal cells, reducing the risk of resistance development. The rapid onset of action is particularly advantageous in therapeutic applications, where minimizing pathogen survival can prevent adaptation and drug resistance.

Previous studies have demonstrated that cationic NCR peptides (*e.g.*, NCR335, NCR247, NCR192) disrupt membrane integrity in *Candida albicans* (Szerencses et al., 2021). In our study, we observed a direct correlation between fungicidal activity and membrane destabilization, evidenced by increased membrane permeability and calcein leakage in peptide-treated *C. neoformans* cells. This suggests that NCR peptides primarily act by disrupting fungal membranes, leading to cytoplasmic leakage and cell death.

Virulence in *C. neoformans* is closely linked to its polysaccharide capsule and melanin synthesis. The negatively charged polysaccharides and melanin deposits contribute to a reduced surface charge, which may impact peptide interaction (Nosanchuk and Casadevall, 1997). Given that antimicrobial peptides rely on electrostatic interactions with negatively charged cell surfaces (Dini et al., 2022), the fungal cell wall and capsule may influence susceptibility. Our findings support this hypothesis, as intact *C. neoformans* cells were more susceptible to NCR peptides than protoplasts, suggesting that the cell wall facilitates peptide binding. This was further supported by calcofluor white experiments, where binding of calcofluor white to the cell wall blocked peptide activity, rendering yeast cells resistant. Additionally, confocal microscopy confirmed that FITC-labelled NCR335 peptides rapidly adhered to the yeast cell surface, highlighting the importance of electrostatic interactions in peptide attachment and activity.

From a clinical perspective, an essential factor in antimicrobial peptide development is host cell safety. Our cytotoxicity assays on J774.2 mouse macrophages confirmed minimal or no toxicity at therapeutically relevant concentrations, supporting the safety profile of NCR peptides for antifungal applications.

Moreover, beyond their direct antifungal action, NCR peptides also enhance macrophage function. Flow cytometry analyses revealed a significant increase in phagocytosis of peptide-treated *C. neoformans* cells, suggesting that NCR peptides may act as opsonins, facilitating yeast engulfment by immune cells. This effect is comparable to the enhanced phagocytosis previously observed with monoclonal antibodies against *C. neoformans* (Mukherjee et al., 1995), reinforcing the potential of NCR peptides as immunomodulatory agents.

## 5 Conclusions

Fungal infections pose a significant global health challenge, necessitating the development of novel antifungal strategies. In this study, we demonstrated the potent antifungal activity of NCR peptide derivatives against *C. neoformans*, highlighting their fungicidal nature, low cytotoxicity, and immune-enhancing properties. We concluded that NCR peptide derivatives effectively kill fungal cells at low concentrations, surpassing standard antifungal drugs in efficacy and their antifungal activity is mediated through membrane disruption, leading to rapid cell death. Moreover, they exhibit no or minimal cytotoxicity toward host cells, making them promising candidates for therapeutic applications. Beyond direct antifungal effects, NCR peptides function as opsonins, enhancing macrophage phagocytosis of fungal cells. The antifungal potency of NCR peptides could be further enhanced by chimeric peptides, underscoring the potential of peptide engineering in drug development.

Our findings underscore the potential of NCR peptides as a novel antifungal strategy with dual direct fungicidal and immune-enhancing effects. Further research into mechanisms of action, *in vivo* efficacy, and pharmacokinetics will be crucial for advancing these peptides toward clinical applications.

## Credit authorship contribution statement

Conceptualization, É.K., G.E. and I.P.; methodology, I.P., G.E., C.P. and A.F.; software, B.S., N.N.; validation, I.P., G.E., C.V. and É.K.; investigation, B.S., A.P., J.S., N.N., C.P. (flow cytometry), A.F. (microscopy); writing, I.P., G.E. and É.K.; visualization, B.S., A.P. and N.N.; editing: I.P., B.S., F.A., G.E. and É.K.; funding acquisition, C.V., F.A. and É.K.; supervision, C.V., G.E.

All authors have read and agreed to the published version of the manuscript.

## Funding

This work was supported by É.K.'s Balzan Prize from the International Balzan Foundation and the Hungarian National Research, Development and Innovation Office Frontline Research grant (KKP129924). F.A was supported by EU’s Horizon 2020 Research and Innovation Program (SGA No. 739593).

## Ethics approval

Not applicable.

## Declaration of generative AI and AI-assisted technologies in the writing process

During the preparation of this work, the authors utilized SciSpace (https://scispace.com/) and ChatGPT 4.0 to enhance the grammatical quality of the manuscript and further refinements, as English is not their native language. Following the use of these AI-assisted tool, the authors carefully reviewed, revised, and edited the content to ensure accuracy, coherence, and alignment with the intended meaning. The authors take full responsibility for the final version of the manuscript and its content.

## Declaration of competing interest

Authors declare that there are no known competing financial interests or personal relationships that could have appeared to influence the work reported.

## Data Availability

Data will be made available on request.

## Appendix Supplementary materials

Supplementary Fig. 1. Growth of *Cryptococcus neoformans* IFM 5844 after AMP-treatment. The applied concentration of the peptides is depicted at the left side while the name of the peptides and the dilution factor of the cell suspension are at the top of the pictures. Image, image 1

Image, application 2

## Footnotes

Supplementary material associated with this article can be found, in the online version, at doi:10.1016/j.crmicr.2025.100407.

## References

[bib0001] Buda De Cesare G., Cristy S.A., Garsin D.A., Lorenz M.C. (2020).

[bib0002] Carvalho Ade O., Gomes V.M. (2009). Plant defensins–prospects for the biological functions and biotechnological properties. Peptides.

[bib0003] Dini I., De Biasi M.G., Mancusi A. (2022). An overview of the potentialities of antimicrobial peptides derived from natural sources. Antibiotics.

[bib0004] Du L., Yang Y., Gu J., Chen J., Liao W., Zhu Y. (2015). Systemic review of published reports on primary cutaneous cryptococcosis in immunocompetent patients. Mycopathologia.

[bib0005] Farkas A., Maroti G., Durgo H., Gyorgypal Z., Lima R.M., Medzihradszky K.F., Kondorosi E. (2014). *Medicago truncatula* symbiotic peptide NCR247 contributes to bacteroid differentiation through multiple mechanisms. Proc. Natl. Acad. Sci. U.S.A..

[bib0006] Farkas A., Pap B., Kondorosi E., Maroti G. (2018). Antimicrobial activity of NCR plant peptides strongly depends on the test assays. Front. Microbiol..

[bib0007] Feng X., Jin S., Wang M., Pang Q., Liu C., Liu R., Liu Y. (2020). The critical role of tryptophan in the antimicrobial activity and cell toxicity of the duck antimicrobial peptide DCATH. Front. Microbiol..

[bib0008] Iyer K.R., Revie N.M., Fu C., Robbins N., Cowen L.E. (2021). Treatment strategies for cryptococcal infection: challenges, advances and future outlook. Nat. Rev. Microbiol..

[bib0009] Jenei S., Tiricz H., Szolomajer J., Timar E., Klement E., Al Bouni M.A., Kondorosi E. (2020). Potent chimeric antimicrobial derivatives of the *Medicago truncatula* NCR247 symbiotic peptide. Front. Microbiol..

[bib0010] Laniado-Laborin R., Cabrales-Vargas M.N. (2009). Amphotericin B: side effects and toxicity. Rev. Iberoam. Micol..

[bib0011] Maroti G., Downie J.A., Kondorosi E. (2015). Plant cysteine-rich peptides that inhibit pathogen growth and control rhizobial differentiation in legume nodules. Curr. Opin. Plant Biol..

[bib0012] Maziarz E.K., Perfect J.R. (2016). Cryptococcosis. Infect. Dis. Clin. North Am..

[bib0013] Montiel J., Downie J.A., Farkas A., Bihari P., Herczeg R., Balint B., Kondorosi E. (2017). Morphotype of bacteroids in different legumes correlates with the number and type of symbiotic NCR peptides. Proc. Natl. Acad. Sci. U.S.A..

[bib0014] Mukherjee S., Lee S.C., Casadevall A. (1995). Antibodies to *Cryptococcus neoformans* glucuronoxylomannan enhance antifungal activity of murine macrophages. Infect. Immun..

[bib0015] Nelson B.N., Hawkins A.N., Wozniak K.L. (2020). Pulmonary macrophage and dendritic cell responses to *Cryptococcus neoformans*. Front. Cell Infect. Microbiol..

[bib0016] Nosanchuk J.D., Casadevall A. (1997). Cellular charge of *Cryptococcus neoformans*: contributions from the capsular polysaccharide, melanin, and monoclonal antibody binding. Infect. Immun..

[bib0017] Olson D.P., Taylor B.J., Ivy S.P. (2001). Detection of MRP functional activity: calcein AM but not BCECF AM as a multidrug resistance-related protein (MRP1) substrate. Cytometry.

[bib0018] Ordogh L., Voros A., Nagy I., Kondorosi E., Kereszt A. (2014). Symbiotic plant peptides eliminate *Candida albicans* both *in vitro* and in an epithelial infection model and inhibit the proliferation of immortalized human cells. Biomed. Res. Int..

[bib0019] Rajasingham R., Govender N.P., Jordan A., Loyse A., Shroufi A., Denning D.W., Boulware D.R. (2022). The global burden of HIV-associated cryptococcal infection in adults in 2020: a modelling analysis. Lancet Infect. Dis..

[bib0020] Sati, H., Beardsley, J., Alastruey-Izquierdo, A., Alffenaar, J.-W., Morrissey, O., Beyer, P., Getahun, H. (2022). *WHO fungal priority pathogens list to guide research development and public health*.

[bib0021] Spadari C.C., Wirth F., Lopes L.B., Ishida K. (2020). New approaches for cryptococcosis treatment. Microorganisms.

[bib0022] Szerencses B., Gacser A., Endre G., Domonkos I., Tiricz H., Vagvolgyi C., Kondorosi E. (2021). Symbiotic NCR peptide fragments affect the viability, morphology and biofilm formation of *Candida* species. Int. J. Mol. Sci..

[bib0023] Tiricz H., Szucs A., Farkas A., Pap B., Lima R.M., Maroti G., Kereszt A. (2013). Antimicrobial nodule-specific cysteine-rich peptides induce membrane depolarization-associated changes in the transcriptome of *Sinorhizobium meliloti*. Appl. Environ. Microbiol..

[bib0024] Tugume L., Ssebambulidde K., Kasibante J., Ellis J., Wake R.M., Gakuru J., Boulware D.R. (2023). Cryptococcal meningitis. Nat. Rev. Dis. Prim..

[bib0025] Velivelli S.L.S., Czymmek K.J., Li H., Shaw J.B., Buchko G.W., Shah D.M. (2020). Antifungal symbiotic peptide NCR044 exhibits unique structure and multifaceted mechanisms of action that confer plant protection. Proc. Natl. Acad. Sci. U.S.A..

[bib0026] Yandek L.E., Pokorny A., Floren A., Knoelke K., Langel U., Almeida P.F. (2007). Mechanism of the cell-penetrating peptide transportan 10 permeation of lipid bilayers. Biophys. J..

[bib0027] Zaragoza O. (2019). Basic principles of the virulence of *Cryptococcus*. Virulence.

[bib0028] Zhang Q.Y., Yan Z.B., Meng Y.M., Hong X.Y., Shao G., Ma J.J., Fu C.Y. (2021). Antimicrobial peptides: mechanism of action, activity and clinical potential. Mil. Med. Res..

[bib0029] Zhao Y., Ye L., Zhao F., Zhang L., Lu Z., Chu T., Wang L. (2023). *Cryptococcus neoformans*, a global threat to human health. Infect. Dis. Poverty.

