# Supplementary figures and images for: Plant-derived nodule-specific cysteine-rich peptides as potent antifungal agents against Cryptococcus neoformans: mechanisms of action, chimeric peptide enhancement, and immunomodulatory effects
Source: Curr Res Microb Sci. 2025 May 23;9:100407. doi: 10.1016/j.crmicr.2025.100407 (PMC12164026; doi:10.1016/j.crmicr.2025.100407)

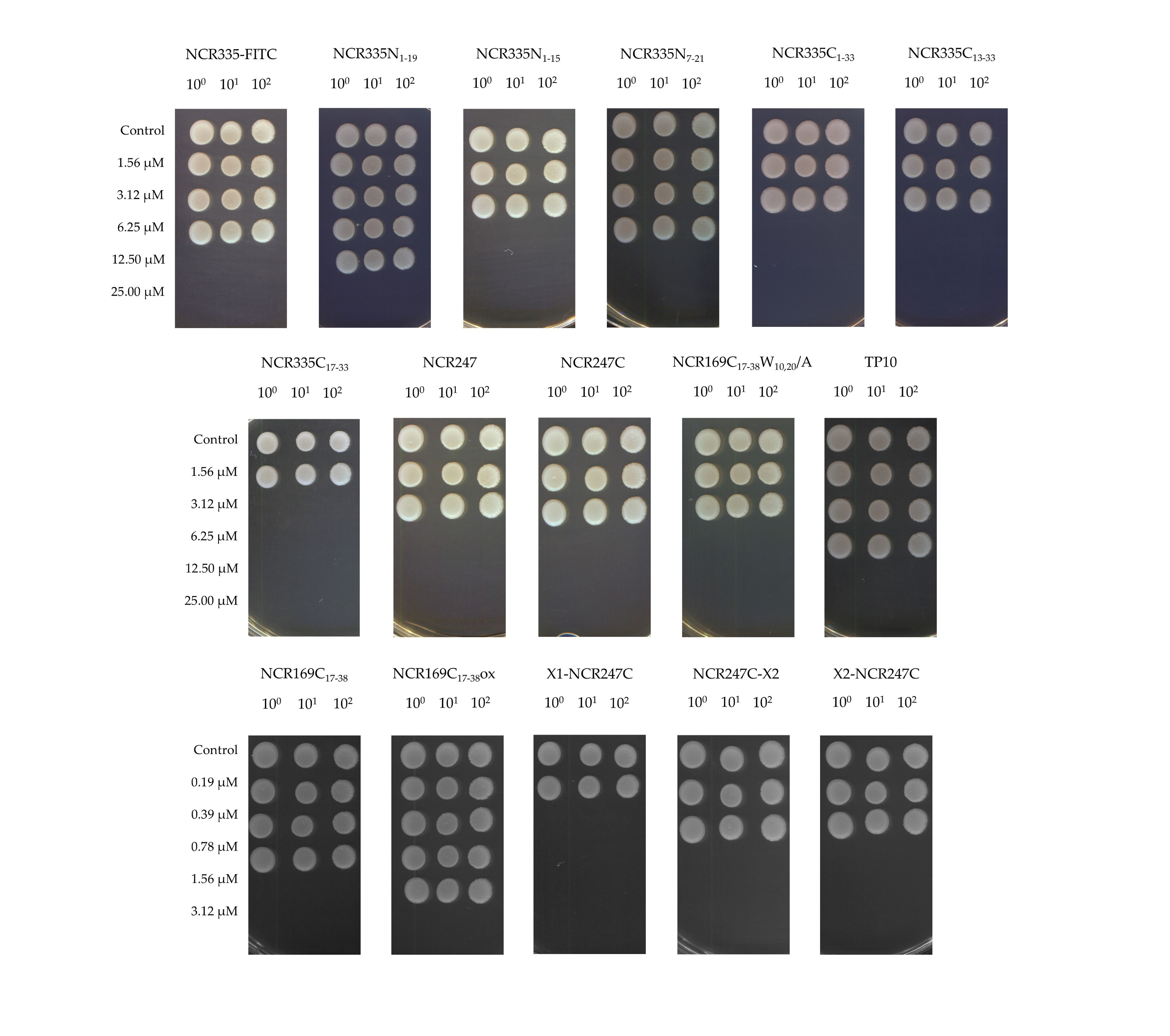

Supplement: Supplementary file 1 — Supplementary Fig. 1. Growth of Cryptococcus neoformans IFM 5844 after AMP-treatment. The applied concentration of the peptides is depicted at the left side while the name of the peptides and the dilution factor of the cell suspension are at the top of the pictures. [file mmc1.jpg]
