# Supplementary material for: Plant-derived nodule-specific cysteine-rich peptides as potent antifungal agents against Cryptococcus neoformans: mechanisms of action, chimeric peptide enhancement, and immunomodulatory effects
Source: Curr Res Microb Sci. 2025 May 23;9:100407. doi: 10.1016/j.crmicr.2025.100407 (PMC12164026; doi:10.1016/j.crmicr.2025.100407)
Supplement: Supplementary file 2 [file mmc2.docx]

**List of the peptides with their physico-chemical properties.**

Peptides were synthetized with C-terminal amidation as it was described in details earlier (Jenei et al., 2020 and Szerencses et al., 2021)

| **Peptides** | **Sequence of peptides** | No. AA | pI | Net Charge |
| --- | --- | --- | --- | --- |
| **NCR169C_17–38_** | KSKKPLFKIWKCVENVCVLWYK | 22 | 10.48 | +5.9 |
| **NCR169C_17–38_ox** | KSKKPLFKIWKĈVENVĈVLWYK | 22 | 11.01 | +6 |
| **NCR169C_17–38_W_10,20_/A** | KSKKPLFKI**A**KCVENVCVL**A**YK | 22 | 10.48 | +5.9 |
| **NCR335** | RLNTTFRPLNFKMLRFWGQNRNIMKHRGQKVHFSLILSDCKTNKDCPKLRRANVRCRKSYCVPI | 64 | 11.22 | +14 |
| **NCR335N_1–19_** | RLNTTFRPLNFKMLRFWGQ | 19 | 14 | +5 |
| **NCR335N_1–15_** | RLNTTFRPLNFKMLR | 15 | 14 | +5 |
| **NCR335N_7–21_** | RPLNFKMLRFWGQNR | 15 | 14 | +5 |
| **NCR335N_16–29_** | FWGQNRNIMKHRGQ | 14 | 14 | +4.1 |
| **NCR335C_1–33_** | HFSLILSDCKTNKDCPKLRRANVRCRKSYCVPI | 33 | 10.37 | +6.8 |
| **NCR335C_13–33_** | KDCPKLRRANVRCRKSYCVPI | 21 | 10.91 | +6.8 |
| **NCR335C_17–33_** | KLRRANVRCRKSYCVPI | 17 | 11.73 | +6.9 |
| **NCR335C_1-8_** | HFSLILSD | 8 | 7.57 | +0.1 |
| **NCR335C_9-16_** | CKTNKDCP | 8 | 9.23 | +1.9 |
| **NCR335C_1-16_** | HFSLILSDCKTNKDCP | 16 | 8.07 | +1 |
| **NCR335C_17-27_** | KLRRANVRCRK | 11 | 12.59 | +6.9 |
| **NCR247** | RNGCIVDPRCPY**QQCRRPLYCRRR** | 24 | 10.82 | +6.7 |
| **NCR247C** | **QQCRRPLYCRRR** | 12 | 11.93 | +5.9 |
| **X1-NCR247C** | RPLNFKMLRFWG**QQQCRRPLYCRRR** | 25 | 12.23 | +8.9 |
| **X2-NCR247C** | KALAALAKKIL**QQCRRPLYCRRR** | 23 | 11.98 | +8.9 |
| **NCR247C-X2** | **QQCRRPLYCRRR**KALAALAKKIL | 23 | 11.98 | +8.9 |
| **X2 (TP10_11-21_)** | KALAALAKKIL | 11 | 14 | +4 |
| **TP10** | AGYLLGKINLKALAALAKKIL | 21 | 11.21 | +5 |

Ĉ indicates two cysteines joined by disulphide-bridge. Alanine residues replacing W_10_ and W_20_ are underlined, the NCR247C sequence is highlighted in each derivative.

AA: amino acids, pI: isoelectric point
